# Dollo Parsimony Overestimates Ancestral Gene Content Reconstructions

**DOI:** 10.1093/gbe/evae062

**Published:** 2024-03-22

**Authors:** Alex Gàlvez-Morante, Laurent Guéguen, Paschalis Natsidis, Maximilian J Telford, Daniel J Richter

**Affiliations:** Institut de Biologia Evolutiva (CSIC-Universitat Pompeu Fabra), Barcelona 08003, Spain; LBBE, UMR 5558, CNRS, Université Claude Bernard Lyon 1, Villeurbanne 69622, France; Centre for Life's Origins and Evolution, Department of Genetics, Evolution and Environment, University College London, London WC1E 6BT, UK; Centre for Life's Origins and Evolution, Department of Genetics, Evolution and Environment, University College London, London WC1E 6BT, UK; Institut de Biologia Evolutiva (CSIC-Universitat Pompeu Fabra), Barcelona 08003, Spain

**Keywords:** ancestral reconstruction, Dollo parsimony, maximum likelihood, gene family evolution, phylogenomics

## Abstract

Ancestral reconstruction is a widely used technique that has been applied to understand the evolutionary history of gain and loss of gene families. Ancestral gene content can be reconstructed via different phylogenetic methods, but many current and previous studies employ Dollo parsimony. We hypothesize that Dollo parsimony is not appropriate for ancestral gene content reconstruction inferences based on sequence homology, as Dollo parsimony is derived from the assumption that a complex character cannot be regained. This premise does not accurately model molecular sequence evolution, in which false orthology can result from sequence convergence or lateral gene transfer. The aim of this study is to test Dollo parsimony's suitability for ancestral gene content reconstruction and to compare its inferences with a maximum likelihood-based approach that allows a gene family to be gained more than once within a tree. We first compared the performance of the two approaches on a series of artificial data sets each of 5,000 genes that were simulated according to a spectrum of evolutionary rates without gene gain or loss, so that inferred deviations from the true gene count would arise only from errors in orthology inference and ancestral reconstruction. Next, we reconstructed protein domain evolution on a phylogeny representing known eukaryotic diversity. We observed that Dollo parsimony produced numerous ancestral gene content overestimations, especially at nodes closer to the root of the tree. These observations led us to the conclusion that, confirming our hypothesis, Dollo parsimony is not an appropriate method for ancestral reconstruction studies based on sequence homology.

**Significance**

Dollo parsimony is a widely used phylogenetic inference method to reconstruct the evolutionary history of gene gain and loss based on genomic data, but it relies on strong assumptions developed for morphological characters that may not be appropriate for sequence data. Using simulated sequence data, we demonstrated that Dollo parsimony consistently overestimates ancestral gene content, with larger overestimates concentrated toward the oldest evolutionary branches; we next showed a similar pattern in real data when reconstructing early eukaryotic evolution. These findings suggest past conclusions based on Dollo parsimony are likely to be at least partially incorrect and, in order to mitigate the effects of methodological biases, Dollo parsimony should be compared with alternative methods to more accurately reconstruct evolutionary history.

## Introduction

Ancestral reconstruction is the inference of ancient characteristics based on extant species characteristics across a phylogenetic tree relating to those species. It can be applied to sequences (DNA, RNA, or protein), as well as to morphological characters. It has been widely used to understand the history of gain and loss of gene families over long timescales and to produce hypotheses on how these gains and losses may have influenced the evolutionary trajectories of extant organisms (Groussin et al. 2016). The power of this technique has allowed it to play a crucial role in diverse topics from tick evolution (Mans et al. 2016) to flower morphology and pollination (Pérez et al. 2006) to the unicellular-to-multicellular transition (Ros-Rocher et al. 2021) and many others (Sverdlov et al. 2004; Kohn et al. 2006; Harms and Thornton 2010).

Ancestral reconstructions of gene family gain and loss can be based on different phylogenetic inference methods, such as maximum likelihood or Bayesian inference, but many current studies are based on Dollo parsimony.

Dollo parsimony is a specific case of maximum parsimony based on Dollo's law (Dollo 1893), which was based on an interpretation of morphological characters and postulates that the same evolutionary path cannot be followed more than once, precluding the possibility that an identical character can be gained twice. This premise is implemented in Dollo parsimony by allowing characters to be gained only once, but accepting as many losses as necessary (Felsenstein 1983).

The literature is replete with examples using Dollo parsimony as a phylogenetic inference method. One of the most frequent applications of Dollo parsimony has been in reconstructing the gains and losses of genes in the lineages leading to major multicellular eukaryotic groups, including land plants (Bowles et al. 2020), animals (Fairclough et al. 2013; Paps and Holland 2018; Najle et al. 2023; Yu et al. 2024), and brown algae (Cock et al. 2010). It has been applied to examine patterns of gene gain and loss in the evolution of novel trophic modes or in the adaptation to specific environments in fungi and their relatives (Mikhailov et al. 2017, 2022; Galindo et al. 2018, 2021), in green algae (Repetti et al. 2022), and in red algae (Cho et al. 2023). Dollo parsimony has also been employed to investigate the evolution of gene gains and losses that may have led to physiological changes such as those underlying the evolution of *Wolffia australiana*, the smallest known flowering plant (Park et al. 2021).

In addition to analyses of gene gain and loss, Dollo parsimony has been applied to infer phylogenies in sweet cherry cultivars (Zhou et al. 2005), in *Mycobacterium* (Stevenson et al. 2002), and, using retroelements, in Laurasiatheria (a group of mammals) (Doronina et al. 2017). Dollo parsimony has also been used to reconstruct protein domain (Zmasek and Godzik 2011) and intron (Csuros et al. 2011) gains and losses across the eukaryotic tree of life and to study inverted repeat region structure, pseudogenization, and gene loss in *Pedicularis*, a hemiparasitic land plant (in comparison with other reconstruction methods) (Li et al. 2021).

The basic assumption of a single gain of an orthologous gene family in Dollo parsimony is also implicit in phylostratigraphy, a widely used approach to reconstruct patterns of gene gain over evolutionary timescales, in which gene origins are assigned to the most recent common ancestor of the extant species in which the gene is found (Domazet-Loso et al. 2007; Domazet-Lošo and Tautz 2010).

In ancestral gene content reconstruction studies, the standard process is first to use an orthology inference program such as OrthoFinder2 (Emms and Kelly 2019), which uses BLAST (or a BLAST-like software) to search for homology among the gene sequences of all input extant species (Altschul et al. 1990). Sequence similarity values are subsequently used as the basis to construct orthologous groups, generating an output (in the case of Dollo parsimony, a binary output representing the presence or absence of an orthologous group in a species) that is used as the input for the ancestral reconstruction programs.

Although Dollo parsimony is a practical method that can be appealingly simple and computationally inexpensive, we hypothesize that it is not appropriate when the input data for ancestral reconstruction are derived from sequence homology. Dollo parsimony operates under the assumption that a feature can only be gained once. Under this assumption, if a gene is present in two different species anywhere in the analyzed phylogeny, it will always be inferred to have been present in their most recent common ancestor, even if the sequence similarity between the genes in the two species may have arisen by chance; the more the two species are distantly related, the more the origin of the gene will be pulled toward the root. This assumption does not take into account convergent sequence evolution (homoplasy) or horizontal gene transfer, and we posit that it results in an overestimation of gene losses and an underestimation of gene gains. Moreover, and even though Dollo parsimony assumptions were developed for morphological characters, Dollo parsimony can still generate distortions in morphological studies, as its assumptions will bias any inference where convergence is possible.

In order to test our hypothesis, we compared the ancestral gene content reconstructions produced by PHYLIP Dollop (Dollo parsimony) (Felsenstein 1983) against Bppancestor (a maximum likelihood method with a model of gene gain and loss in order to assess ancestral presence) (Guéguen et al. 2013) for a simulated data set based on a phylogeny of metazoans. This data set contained 200 independent simulations of the evolution of protein sequences over a fixed topology of 57 animal species (Natsidis et al. 2021). Each of these simulations contained 5,000 orthologous groups that were present in all 57 species, with no gains or losses allowed.

Next, we compared the reconstruction of Pfam protein domain evolution across the eukaryotic tree of life produced by Dollo parsimony versus maximum likelihood. We used the insights gained from our analysis of simulated data to compare the results of this reconstruction to a previous study, based on Dollo parsimony, which found that protein domain loss outweighed protein domain gain across eukaryotes and that the last eukaryotic common ancestor (LECA) possessed a protein domain repertoire larger than any extant species (Zmasek and Godzik 2011).

## Results

### Dollo Parsimony Overestimates Ancestral Gene Content in a Simulated Data Set

We tested the performance of Dollo parsimony on a data set containing 200 simulations of protein sequence evolution on a fixed topology of 57 species (Natsidis et al. 2021). Each of these simulations contained exactly 5,000 orthologous genes present in all 57 species, with no gains or losses allowed. The 200 simulations differed from each other in their rates of substitution and in the variation of rates among sites within each gene. Separately for each individual simulation, OrthoFinder2 (Emms and Kelly 2019) was run in order to partition simulated gene sequences into orthologous groups (Natsidis et al. 2021).

After ancestral reconstruction, any ancestral nodes inferred to have contained either more than 5,000 genes or fewer than 5,000 genes would represent incorrect estimates of the number of orthologous groups used as input to ancestral reconstruction, in the ancestral reconstruction inference method itself, or both.

We began by examining the contents of the orthogroups to be used as input to ancestral reconstruction. The expected result from a correct partitioning of simulated gene sequences into orthologous groups would be 5,000 orthologous groups, each containing exactly 57 sequences (one from each species). The orthogroups for most simulations contained, on average, sequences from fewer than 57 species (supplementary fig. S1, Supplementary Material online). As a consequence, there were more than the expected 5,000 orthogroups per simulation (supplementary fig. S2, Supplementary Material online), and each of those orthogroups contained only a subset of the 57 species. Most species did not have a gene present in many orthogroups due to the artificial expansion of the number of orthogroups, the generation of singletons (which are not part of any orthogroup in OrthoFinder2's output) and the grouping of multiple genes from the same species in the same orthogroup, which together resulted in an underestimated input value (fewer than 5,000 orthogroups) for most input nodes in most simulations (supplementary fig. S3b, Supplementary Material online). Although simulations with slower rates of evolution were generally correctly partitioned into complete orthologous groups, as the simulated rate of evolution increased, so did the underestimation in the number of orthogroups present in each input species. This effect is likely the result of higher sequence divergence making it less likely that genes could be correctly grouped by homology into orthogroups. Since the simulated rates of evolution span the values likely to be present in real data sets (Natsidis et al. 2021), the composition of orthologous groups we used as input to ancestral reconstruction should be reflective of the scope of potential underestimates present in real data sets.

In order to determine whether Dollo parsimony performs similarly to other ancestral reconstruction methods that do not share its strict assumptions, we analyzed the same data set with a maximum likelihood method, Bppancestor (Guéguen et al. 2013). To exclude the possibility that our particular choice of maximum likelihood software might influence our results, we compared Bppancestor to another implementation, Mesquite (Maddison and Maddison 2023) on a subset of the input data. Bppancestor and Mesquite produced nearly identical ancestral reconstructions (supplementary fig. S4, Supplementary Material online). As Bppancestor can be easily automated and Mesquite cannot, we continued with Bppancestor for further analyses on the full data set.

**Fig. 1.**
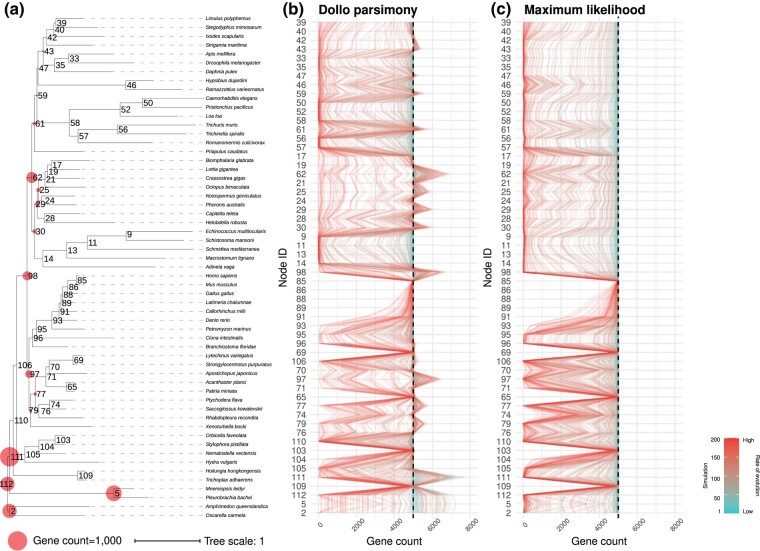
Ancestral gene content reconstructions on a simulated data set containing exactly 5,000 orthologs. a) Phylogenetic tree (Natsidis et al. 2021) depicting the relationship among species used in the simulations, highlighting ancestral nodes that were overestimated by Dollo parsimony in at least one case (circles). The size of the circles is proportional to the largest number (among all simulations) of estimated genes exceeding 5,000. Internal nodes are identified by numbers, which correspond among panels. b) Gene counts at internal nodes inferred by Dollo parsimony. c) Gene counts at internal nodes inferred by maximum likelihood. In b) and c), each line represents the set of inferences from one simulation. Simulation numbers correspond to the rate of sequence evolution used to produce simulated data (lower numbers have lower rates).

**Fig. 2.**
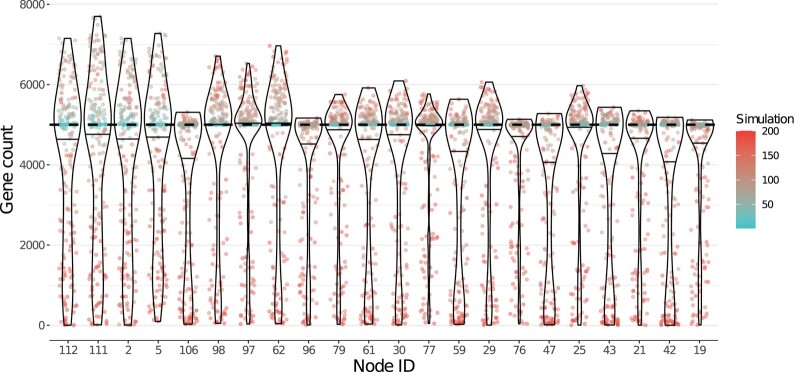
Distributions of inferred gene counts at ancestral nodes that showed the highest levels of overestimation by Dollo parsimony. Each point in a distribution represents the ancestral gene count inferred from one simulation. Simulation numbers correspond to the rate of sequence evolution used to produce simulated data (lower numbers have lower rates). The color scale is identical to that of Fig. 1. The nodes on the horizontal axis are ordered by their proximity to the root of the topology (nodes that are closer to the root appear toward the left). The proximity to the root is measured as the number of internal nodes between the node of interest and the root of the tree. This figure shows nodes that were overestimated by at least 105 genes for at least one simulation; distributions for all internal nodes are shown in supplementary fig. S5, Supplementary Material online.

Dollo parsimony consistently produced reconstructions of ancestral node gene content that were above the true 5,000 genes threshold (Fig. 1a and b). This effect was amplified at nodes closer to the root, where there were more overestimated nodes, and where the estimated gene counts showed the largest inflations above 5,000 (Fig. 2; supplementary fig. S5, Supplementary Material online). The increased overestimates closer to the root that we observed are consistent with our expectations, as nodes closer to the root have more children and thus more opportunities for nonorthologous sequence homology between pairs of distantly related species to arise, which would then be incorrectly inferred by Dollo parsimony to have been present in their most recent common ancestor. In contrast, maximum likelihood never produced estimated counts above the true 5,000 genes threshold (Fig. 1c).

For Dollo parsimony, both relatively slow and relatively fast-evolving simulations produced overestimations (Figs. 1b and 2). For maximum likelihood, inferences from slow-evolving simulations were generally close to the true gene count, whereas fast-evolving simulations resulted in larger distortions but never produced any overestimation. The low estimates that Bppancestor produced with fast-evolving simulations could be explained by the already underestimated input. Areas of the topology with an accurate input count generated more accurate inferences than areas of the topology with a distorted (underestimated) input count (supplementary fig. S3, Supplementary Material online).

### Dollo Parsimony Produces Substantially Higher Estimates of Pfam Domain Content in the Earliest Eukaryotes

To contrast the inferences of Dollo parsimony and maximum likelihood on a real data set, we chose to return to an analysis first carried out in 2011 by Zmasek and Godzik (Zmasek and Godzik 2011). Their aim was to reconstruct the evolution of Pfam protein domain content in eukaryotes, by sampling the available genomes of a diversity of extant species and applying Dollo parsimony to reconstruct the history of domain gain and loss. We repeated their analysis with an updated set of species from EukProt v3 (Richter et al. 2022) and compared the results of Dollo parsimony versus maximum likelihood on the set of Pfam domains annotated to be present in each species.

**Fig. 3.**
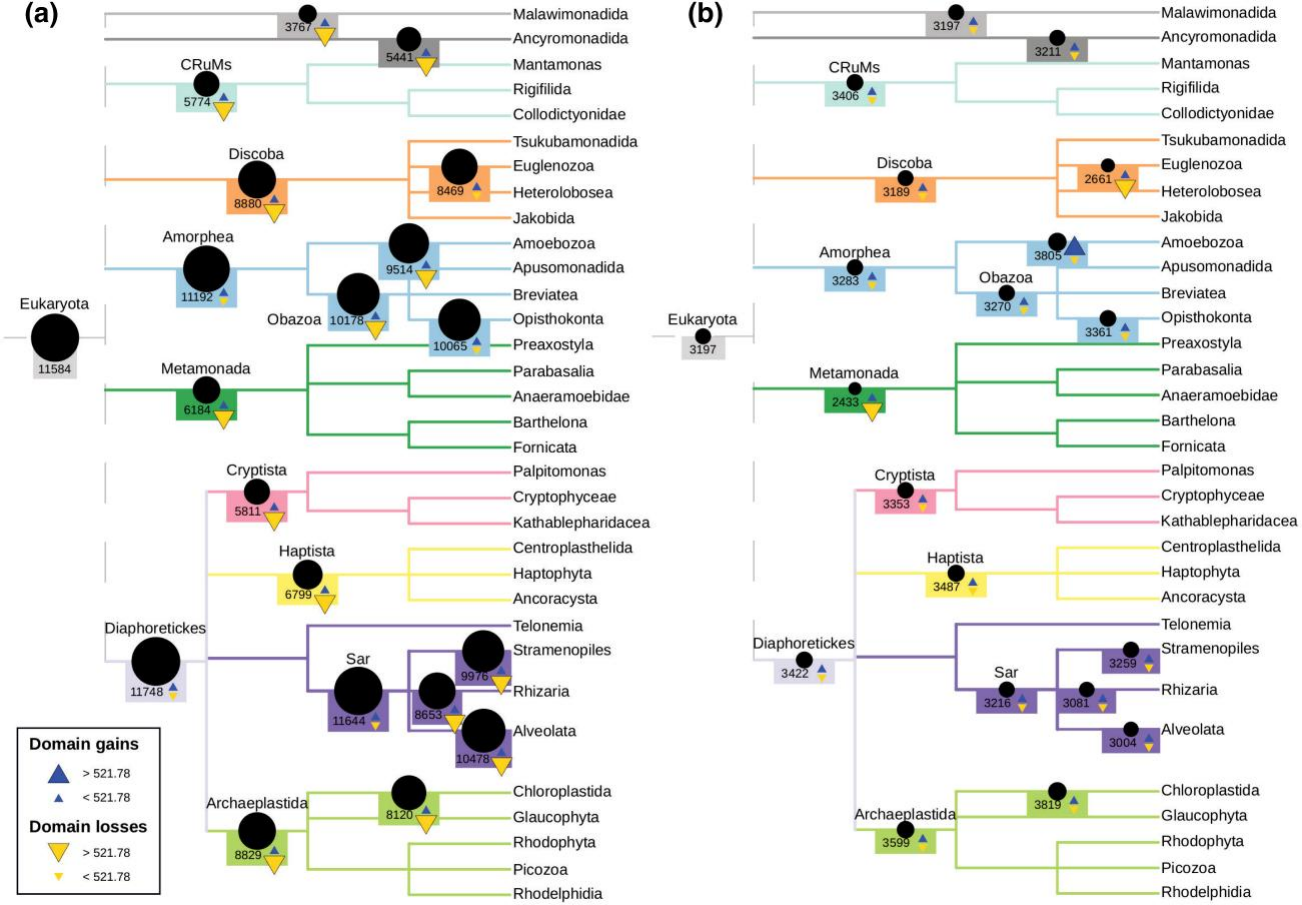
Pfam protein domain counts, gains, and losses during eukaryotic evolution, inferred by a) Dollo parsimony and b) maximum likelihood. The sizes of the circles are proportional to the estimated count of domains present at selected nodes. Upward-pointing triangles represent inferred protein domain gains, while downward-pointing triangles represent inferred protein domain losses. The threshold separating the two different sizes of triangles is derived from the third quartile of all gain and loss inferences (Q3 = 521.78). Numeric counts of gains and losses are shown in supplementary fig. S6, Supplementary Material online. Tree topology and node names are derived from UniEuk (Berney et al. 2017).

Dollo parsimony and maximum likelihood produced substantially different estimates of Pfam domain content at ancestral nodes, as well as counts of domain gain and loss across the eukaryotic tree (Fig. 3). Dollo parsimony produced much larger domain counts than maximum likelihood. The estimates from Dollo parsimony also increased in size with proximity to the root, similar to what we observed in our analysis of simulated data. In fact, Dollo parsimony reconstructed a LECA with a higher Pfam domain content than any extant eukaryote, which represents almost two times the domain content of the highest estimate from maximum likelihood at any ancestral node. Dollo parsimony displayed a clear tendency toward domain loss versus domain gain (45,723 total losses against 872 total gains). In contrast, the results from maximum likelihood were more balanced between domain gain and domain loss (3,706 total losses against 4,829 total gains) (Fig. 3 and supplementary fig. S6, Supplementary Material online). We also observed a major difference regarding where domain gains occurred in the tree: in Dollo parsimony, most of the domain gains are inferred close to the LECA (as we can see in clades such as Diaphoretickes), whereas maximum likelihood infers most of the domain gains closer to the leaves of the tree (as can be seen in clades such as Amoebozoa) (supplementary fig. S6, Supplementary Material online).

Our Dollo parsimony inferences are coherent with the results obtained by Zmasek and Godzik. Both studies produced a loss-dominated reconstruction of eukaryotic evolution, with relatively few exceptions. A large protein domain repertoire in the LECA, larger than any extant species, was also inferred in both studies. Even though these two inferences coincide, they are inconsistent with our maximum likelihood reconstruction, which displayed a balance between domain gain and loss and inferred a LECA with a smaller number of unique Pfam domains than many extant species.

## Discussion

In this study, we tested the hypothesis that Dollo parsimony overestimates ancestral gene content reconstructions, by using simulated data as an input. Next, we analyzed a real data set of Pfam protein domain content across eukaryotes with Dollo parsimony and with maximum likelihood, which did not show evidence for overestimations on simulated data.

The use of simulated data without gene gains or losses, in the first section of this study, provided Dollo parsimony with a “favorable” scenario where no convergent gains, nonorthologous homology (e.g. gene families with ancient duplications), or horizontal gene transfers could generate overestimations in inferred ancestral gene counts. Moreover, the gene counts of extant species provided as input were substantially underestimated for the fastest-evolving simulations.

Even in this favorable scenario for Dollo parsimony, we found a clear tendency of Dollo parsimony to overestimate both gene content, as many values were inferred to be higher than the true ancestral value, and gene loss, as the overestimations were larger toward the root of the tree topology. These overestimations result only from orthology inference errors (the splitting of the original orthogroups and random sequence similarity) and could be much more pronounced in a real case, where secondary gains, non-orthologous homology, and horizontal gene transfer play a role.

The results of Dollo parsimony were more accurate on slower versus faster-evolving simulations. In faster-evolving simulations, true orthologs were split across multiple orthogroups in Dollo's input data, resulting in overestimated inferences, as ancestral nodes contained artificially generated orthogroups in addition to the true ones (supplementary fig. S2, Supplementary Material online). Beginning with a larger number of input orthogroups in an ancestral reconstruction performed with Dollo parsimony increases the potential number of inferred genes at ancestral nodes. If multiple artificially generated orthogroups for a single group of true orthologs each contain representative genes from phylogenetically distant species, then inflation of ancestral counts would occur at those species’ last common ancestors. Although this aspect of the input data led to inflation in Dollo parsimony's ancestral gene content estimates, not all aspects are likely to lead to inflated estimates. In fact, two other aspects would be expected to produce reductions. First, with increasing evolutionary rates, the likelihood also increases that simulated sequences are so distant that they are excluded entirely from any orthogroup (i.e. they are singletons); this would lead to the associated genes being considered to be absent in the species. Second, Dollo parsimony's input data are binary (either present or absent); therefore, we only accounted for the presence or absence of any sequence from a given orthogroup in a species’ proteome, not the number of sequences. When multiple sequences from the same species were incorrectly partitioned into the same orthogroup, they would only be counted once, thereby reducing the total number of input gene presences.

Nonneutral evolution could accentuate the distortions observed in this study, with a greater impact in deep nodes, as seen in previous work (Holland et al. 2020). Cases where evolution is indeed directional and where the ancestral state is unfavored, either by extinction rate (state-dependent likelihood of species extinction) or state-change asymmetry (unequal state-dependent probability of transition between states), will suffer greater biases, as it is an evolutionary scenario prone to both a large amount of transitions and to convergence, which conflict with Dollo parsimony assumptions. In addition, studies focused on ancestral sequence inference (rather than on gain and loss of gene families) found similar observations to ours (Zhang and Nei 1997): a higher amino acid sequence divergence resulted in a reduction of accuracy in both maximum likelihood and Dollo parsimony methods, with an overall better accuracy of maximum likelihood over Dollo parsimony across different levels of sequence divergence.

Although Dollo parsimony produced estimates of ancestral gene content that were above the input value of 5,000 in many cases, its inferences in other cases were not inflated above 5,000 and were closer than those of maximum likelihood to the true input value (supplementary fig. S7, Supplementary Material online). We believe that this is an artifact of the input data to ancestral reconstruction, as the underestimated inferences of maximum likelihood are expected given the underestimated input (supplementary fig. S3, Supplementary Material online), which is caused by the inappropriate generation of singletons and the incorrect grouping of multiple genes from the same species in the same orthogroup. Dollo parsimony generally produced overestimated inferences across our data set. When presented with underestimated input values, it produced ancestral reconstructions that were overestimates of these underestimates. Therefore, although Dollo parsimony generated clear overestimations, it might have the effect of compensating for underestimations in input data for some cases of ancestral reconstruction.

Our results also have implications for the phylostratigraphy approach, which also relies on Dollo parsimony's assumption that a gene may only be gained once over evolutionary history, although we note that both gene age overestimation (as might be expected to occur given our results) and gene age underestimation within the phylostratigraphy approach have been extensively debated in the literature (Moyers and Zhang 2015, 2016, 2017, 2018; Domazet-Lošo et al. 2017; Casola 2018).

We note that our study is based on a single tree topology, which is the one used to generate the simulations. Nonetheless, our results should be generalizable to other tree topologies, as they result from the underlying assumptions of either Dollo parsimony or maximum likelihood. In general, the tree size (total number of tips) should be positively correlated with Dollo parsimony distortions, as the more tips, the more possibility of artificial orthology matches between species.

In the second part of our study, we observed strikingly different estimates of ancestral eukaryote Pfam protein domain content when reconstructed with Dollo parsimony versus maximum likelihood. These contradictory results indicate that conclusions of evolutionary studies based on ancestral reconstruction can be extremely dependent on the methodology used.

Our ancestral reconstruction using Dollo parsimony inferred that the LECA had more Pfam domains than any extant eukaryote and that the evolutionary history of Pfam domains in eukaryotes was dominated by loss. These results are consistent with those of a previous study based on a smaller number of data sets available at the time (Zmasek and Godzik 2011) and may either reflect the true evolutionary history of Pfam domains in eukaryotes or may be the result of distortions due to Dollo parsimony. The fact that we demonstrated Dollo parsimony's inherent tendency to overestimate both ancestral gene content and the number of gene losses using simulated data as input suggests that the evolutionary scenario inferred by Dollo parsimony may have been an artifact of the methodology that was applied. A single-origin model of evolution has already been proven to support the “genome of Eden hypothesis,” which posits a last common eukaryotic ancestor with an enormous range of genomic content, essentially consisting of any gene that is now seen in at least two major eukaryotic groups (Doolittle et al. 2003). Allowing for multiple gains across the eukaryote tree, to allow for the possibility of horizontal gene transfer and other processes, could offer a potential solution to mitigate Dollo parsimony's inclination to overestimate ancestral gene content (Dagan and Martin 2007).

In the context of our analysis, it is relevant to remark that Pfam profile HMMs are derived from a representative alignment of select taxa, which results in a biased protein domain detection toward biomedically relevant species and an underestimation of detected domains in nonmodel species (Tassia et al. 2021). Another possible bias in our inference might be caused by the heuristic E-value thresholds implemented in InterProScan (Jones et al. 2014) to assess protein domain presence or absence in input species’ proteomes. These phenomena may help explain the relatively low estimates of domain presence and high numbers of domain losses inferred by both ancestral reconstruction methods in groups that are poorly represented in protein sequence databases (e.g. Metamonada).

Dollo parsimony, and other phylogenetic inference methods and programs involved in the process of ancestral reconstruction, induce biases in the inference of gene content. Therefore, we propose that, in order to mitigate the effects of these biases, the results of different methods should be contrasted in order to assess which ancestral reconstructions are more likely to be an accurate representation of the evolution of the studied organisms rather than an artifact of the methodology. Some alternatives to Dollo parsimony that could be used and compared with each other in ancestral reconstruction studies are programs such as Bppancestor and Mesquite (maximum likelihood) (Guéguen et al. 2013; Maddison and Maddison 2023), Count (Wagner parsimony and linear birth–death–immigration method) (Csűös 2010), or MrBayes (Bayesian inference) (Ronquist et al. 2012). Gene tree/species reconciliation methods, such as ALE (Szöllősi et al. 2013, 2015), could also enhance these analyses, as they can also detect horizontal gene transfer events. The orthology inference performed by OrthoFinder2 (Emms and Kelly 2019) also added some degree of distortion to our input data. The usage of a more sensitive method (ideally without an accompanying loss of specificity) would help detect more divergent orthologs, which would alleviate these distortions. Alternative orthology inference methods such as Broccoli (mixed phylogeny-network approach) (Derelle et al. 2020) could be used as valuable comparisons with OrthoFinder2.

Overall, our results indicate that, in ancestral reconstruction studies based on sequence homology, Dollo parsimony tends to overestimate both ancestral gene content and gene loss; consequently, the results of different phylogenetic inference methods should be compared in order to obtain a coherent portrait of evolutionary history. We also suggest that, for the purpose of improving future studies based on ancestral reconstructions, efforts could be focused on producing more accurate orthology inference methods, as ancestral gene content reconstruction methods will always depend on the input data.

## Methods

### Simulated Data Set: Input Data

A total of 200 data sets derived from simulations of the evolution of protein sequences for a fixed topology of 57 species, representing metazoan phylogeny, were obtained from Natsidis et al. (2021). Each of these simulations contained 5,000 sets of orthologs present in all 57 species, and no gains or losses were allowed during the evolution of the protein sequences. The simulation experiments were performed in artificial life framework (ALF) (Dalquen et al. 2012), using parameter values derived from empirical data. Each of the 200 simulations differed from each other in their overall evolutionary rates. Guide tree branch lengths were multiplied by a randomly chosen scalar between 0.2× and 10×, and a single alpha parameter for rate variation among sites was derived for each simulation from an empirically derived distribution (from 0.4 to 1.6). Sequence evolution of each of the 5,000 genes was simulated independently along the guide tree with the LG model.

OrthoFinder2, a platform for comparative genomics (Emms and Kelly 2019), was run by Natsidis et al. separately on all 200 sets of 285,000 protein sequences (5,000 protein sequences per species). OrthoFinder's output was converted with Perl scripts into a binary format that was used as input both for Bppancestor and for PHYLIP Dollop, in which each orthogroup in each species was scored as either present or absent. The version used was Perl 5.30.0 (Wall et al. 2000).

### Simulated Data Set: Running Bppancestor

Using Perl scripts, we generated one configuration file per each input file, using a template (template_bppancestor_config_file.conf, available in the GitHub repository). The configuration files specified a stationary process with a binary birth/death model and a gamma distribution of rate variation among sites (with default parameter values), which was stated to remain homogeneous across all branches of the topology. We performed tests with estimated parameters on two randomly selected simulations with different rates, which generated equivalent results (supplementary fig. S8, Supplementary Material online), and tests with a nonstationary model on the same two simulations, which produced identical results.

We ran Bppancestor iteratively to perform the ancestral reconstructions on each simulation and processed the results with Perl scripts to parse their results. The number of presences at each node was counted by treating the estimated probabilities as expected values and summing them across all sites. The used versions were Bio++ version 3.0.0 (Guéguen et al. 2013) and Perl 5.30.0 (Wall et al. 2000).

### Simulated Data Set: Running PHYLIP Dollop

We ran PHYLIP Dollop separately for each simulation. We used PHYLIP Dollop's default options, except for the option “Search for the best tree,” which we disabled because we provided a fixed tree topology as input, and the option “Print States at all nodes,” which we enabled. All operations were performed under PHYLIP version 3.697 (Felsenstein 1983).

### Simulated Data Set: Running Mesquite

The phylogenetic analysis was carried out using the “Trace All Characters” option in the “Tree:Analysis” tab of the “Tree Block” section of the program, with default settings and inputting an initial states file and a fixed phylogenetic tree. The initial states file was inputted in Nexus format, while the tree file was inputted in Newick format. All the operations were performed under Mesquite version 3.61 (Maddison and Maddison 2023).

### Pfam Domain Content in the Earliest Eukaryotes: Input Data

A data set containing 993 protein sets representing eukaryotic diversity was obtained from EukProt v3 (Richter et al. 2022). Then, we ran InterProScan 5.56-89.0 (Jones et al. 2014) to detect Pfam domains in the protein sequences. We converted the results into a binary format that was used as input both for Bppancestor and for PHYLIP Dollop, indicating the presence or absence of each Pfam domain in each species. The version used was Perl 5.30.0 (Wall et al. 2000).

### Pfam Domain Content in the Earliest Eukaryotes: Tree Topology

We ran Gappa 0.8.2 (Czech et al. 2020) on the taxonomy obtained from EukProt v3 (Richter et al. 2022) in order to generate our initial input tree. We used the AfterPhylo perl script (Zhu 2014) to truncate the names of the tree to 10 characters and resolved it using the “multi2di” R function, from R version 3.6.3 (R Core Team 2022), in order to generate the final version of the tree for PHYLIP Dollop. Multiple different versions of the randomly resolved tree were tested and generated equivalent results (data not shown).

### Pfam Domain Content in the Earliest Eukaryotes: Running Bppancestor

To estimate branch lengths and model parameters, we ran Bppml (Guéguen et al. 2013), with a configuration file (template_bppml_config_file.conf, available in the GitHub repository), specifying a stationary process with a binary model and a gamma distribution of rate variation among sites, which was stated to remain homogeneous across all branches of the topology.

We also generated a configuration file for Bppancestor (domains_configuration_file.conf, available in the GitHub repository) with the most likely model estimated by Bppml (binary model with kappa = 0.20 and gamma distribution with alpha = 0.46).

We ran Bppancestor with this configuration file and treated the output with a Perl script to parse the results and count the number of gene gains and losses. The script sums the probabilities of the genes present at each node, treating each individual probability as an expected value of gene presence. It then compares the probability of each gene's presence relative to the parent node. If the probability of presence in the child is higher than the probability in the parent, it is considered as a gain and added to the sum of gains leading to the node; if the probability is lower, then it is considered as a loss and added to the sum of losses leading to the node. The used versions were Bio++ version 3.0.0 (Guéguen et al. 2013) and Perl 5.30.0 (Wall et al. 2000).

### Pfam Domain Content in the Earliest Eukaryotes: Running PHYLIP Dollop

We ran PHYLIP Dollop and treated the output with a Perl script to parse the results and count the number of gene gains and losses. The script sums the number of genes estimated to be present at each node. If there is a change in gene presence relative to the parent, it is recorded as either a gain or a loss. The phylogenetic analysis was carried out using PHYLIP Dollop's default options, except for the option “Search for the best tree,” which we disabled because we provided a fixed tree topology as input, and the option “Print States at all nodes,” which we enabled. All operations were performed under PHYLIP version 3.697 (Felsenstein 1983) and Perl 5.30.0 (Wall et al. 2000).

### Graphics and Figure Design

We used Rstudio 2023.3.0.386 (RStudio Team 2020), iTOL 6 (Letunic and Bork 2007), and Inkscape 1.1.1 (Inkscape Project 2020) to produce and modify figures and phylogenetic trees.

## Supplementary Material

evae062_Supplementary_Data

## Data Availability

Configuration files for Bppancestor and Bppml, as well as Perl scripts, phylogenetic trees and input files used for ancestral reconstructions, are available on GitHub at https://github.com/beaplab/Ancestral-Reconstruction.
